# Cytolethal distending toxin from *Glaesserella parasuis* induces ferroptosis in porcine alveolar macrophages and mice

**DOI:** 10.1186/s13567-025-01520-0

**Published:** 2025-04-25

**Authors:** Shiyu Xu, Li Lei, Zhen Yang, Yu Wang, Senyan Du, Qin Zhao, Xiaobo Huang, Sanjie Cao, Rui Wu, Yiping Wang, Qigui Yan, Yiping Wen

**Affiliations:** 1 Research Center for Swine Diseases, College of Veterinary Medicine, Sichuan Agricultural University, Chengdu, 611130 Sichuan China; 2 Sichuan Science-Observation Experimental Station of Veterinary Drugs and Veterinary Biotechnology, Ministry of Agriculture and Rural Affairs, Chengdu, 61130 China; 3 National Center of Technology Innovation for Pigs, Chongqing, 402460 China; 4 Chongqing Academy of Animal Sciences, Chongqing, 402460 China

**Keywords:** *Glaesserella parasuis*, *Gp*CDT, ferroptosis

## Abstract

*Glaesserella parasuis* cytolethal distending toxin (*Gp*CDT) is a bacterial genotoxin whose main action is to activate DNA damage responses, induce cell cycle arrest, and induce the apoptosis of host cells. In our previous studies, we reported that cells incubated with *Gp*CDT exhibited changes in the expression of ferroptosis-related proteins; thus, we hypothesized that, in addition to apoptosis, *Gp*CDT may also cause ferroptosis, a novel mode of cell death. Here, we observed that treatment of 3D4/21 cells with *Gp*CDT resulted in cytoplasmic iron overload, depletion of GSH (reduced glutathione), and overproduction of reactive oxygen species (ROS) and malondialdehyde (MDA), indicating that *Gp*CDT disrupted iron metabolism and redox homeostasis in these cells. These phenomena were counteracted by the specific ferroptosis inhibitor ferrostatin-1 and the iron chelator deferoxamine mesylate. In vitro infection with the *Glaesserella parasuis* field isolate strain SC1401 (CDT positive) induced changes in the expression of ferroptosis biomarkers and proteins. Infection of C57BL/6 mice yielded similar results. Our results suggest that ferroptosis may play a substantial role in *Gp*CDT-induced cellular injury.

## Introduction

*Glaesserella parasuis* (*G. parasuis*) is a Gram-negative opportunistic pathogen that colonizes the upper respiratory tract of pigs [1]. When the body is stressed, has low immunity, or is secondarily infected, *G. parasuis* may spread to the lungs, causing pneumonia, and may enter the bloodstream, causing systemic Glässer’s disease. Glässer’s disease, characterized by fibrous polyserositis, arthritis, and meningitis, results in significant economic losses to the pig industry worldwide [2]. Vaccination and antibiotic therapy are the most widely utilized methods for preventing and treating *G. parasuis* infection in pigs. However, because of the low degree of cross-protection from vaccines and increased antibiotic resistance of *G. parasuis*, it is increasingly important to identify additional methods for prevention and treatment [3].

Cytolethal distending toxin (CDT) is a heat-labile bacterial genotoxin discovered in 1988 in culture filtrates of *Escherichia coli* (*E. coli*) [4]. It is found mainly in Gram-negative microaerophilic bacteria and plays an important role in the pathogenesis of these bacteria. CDT has DNase activity and causes genotoxicity by inducing double-strand breaks, which lead to cell cycle arrest in a broad range of mammalian cells [5]. To date, CDT has been shown to trigger cell death by apoptosis, pyroptosis, and autophagy [6–8]. *G. parasuis* CDT induces p53-dependent apoptosis in the tracheal cells of neonatal piglets, *Campylobacter jejuni* CDT induces GSDME-dependent pyroptosis in colon epithelial cells, and *Haemophilus ducyrei* CDT induces autophagy in colorectal carcinoma cells [6, 9, 10]. These data indicate that CDT is most likely an important virulence factor of *G. parasuis* and suggest that a more comprehensive and in-depth study of its pathogenic role would be worthwhile.

Ferroptosis is a non-apoptotic type of regulated cell death that differs from autophagy, apoptosis, and necrosis at the biochemical, morphological, and genetic levels. Ferroptosis is characterized by iron overload, the production of reactive oxygen species (ROS), and the accumulation of lipid peroxides [11]. The effects include depletion of glutathione, diminished glutathione peroxidase activity, and ineffective metabolism of lipid oxides by the GPX4-catalyzed glutathione reductase reaction [12]. GPX4 prevents cell membrane disruption and cell death by reducing phospholipid hydroperoxides to nonreactive phosphatidyl alcohols [13]; thus, dysfunctional GPX4 or a reduction in expression renders cells susceptible to lipid peroxidation and ferroptosis [14].

Intracellular iron homeostasis is predominantly regulated post-transcriptionally by iron metabolism-related genes through iron-responsive elements (iron-regulated protein systems), such as ferritin (heavy chain FTH1 and light chain FTL). Ferritin is involved mainly in the storage and release of iron ions, which regulate the intracellular iron concentration and lipid peroxidation levels [15].

ACSL4, an enzyme that converts fatty acids to fatty acyl coenzyme A esters, regulates lipid biosynthesis. Doll et al. demonstrated that ACSL4 is a key player in ferroptosis, using a CRISPR-based genetic screen and analysis of resistant cells [16].

Previous studies on ferroptosis have focused on oncological diseases [17], neurodegenerative diseases [18], and ischemic injuries [19], but in recent years, the role of ferroptosis in pathogenic bacteria‒host cell interactions has received increased attention. Ferroptosis plays a major role in the cell death induced by *Mycobacterium tuberculosis* [20], and *E. coli* can initiate ferroptosis in the erythrocytes of grass carp [21]; however, there have been no reports on the association of *G. parasuis* with ferroptosis.

3D4/21 cells are porcine alveolar macrophages. Macrophages are a type of immune cell in the innate immune system and are widely present in nearly all tissues [22]. They mediate inflammation and regulate iron, lipid, and amino acid metabolism through their unique functions of phagocytosis and cytotoxicity at different polarizations, cytokine secretion and ROS production [23]. The characteristics of ferroptosis and the functions of macrophages have many commonalities [24]; therefore, we chose 3D4/21 cells for use in this study. This study aimed to elucidate the molecular mechanisms by which *G. parasuis* cytolethal distending toxin (*Gp*CDT) regulates programmed cell death, thereby enhancing our understanding of host‒pathogen interactions. Through in vivo and in vitro experiments, we demonstrated for the first time that *Gp*CDT can induce ferroptosis in host cells. This discovery will not only expand our understanding of the biological functions of *Gp*CDT but also reveal its critical role in the pathogenic process of *G. parasuis*. Furthermore, this study provides new insights into the mechanisms of cell death induced by gram-Gram-negative bacterial toxins and lays a solid theoretical foundation for the development of novel antibacterial strategies and immunotherapeutic targets based on the regulation of ferroptosis. These findings hold significant scientific and practical value for the prevention and control of *G. parasuis* infections and related diseases.

## Materials and methods

### Cells and plasmids

3D4/21 cells were grown in RPMI 1640 (Gibco, USA) supplemented with 10% foetal bovine serum (Gibco) in a 37 ℃ humidified 5% CO_2_ atmosphere. The cells were subcultured upon reaching 90% confluence.

All plasmids and bacterial strains used are listed in Table 1. SC1401 is a wild-type strain of *G. parasuis* that is positive for CDT and is classified as serotype 11 [25]. SC1401Δcdt is a CDT-deficient mutant strain derived from SC1401, which was constructed through homologous recombination to completely delete both copies of the *CDT* gene from the genome [26]. The bacterial strains were cultivated at 37 °C in either tryptic soy broth (TSB; BD-Difco, USA) or tryptic soy agar (TSA; BD-Difco, USA), both supplemented with 5% newborn bovine serum (Solarbio, China) and 0.1% (wt/vol) nicotinamide adenine dinucleotide (NAD; Sigma‒Aldrich, USA). For liquid cultures, the strains were maintained under continuous shaking at 220 r/min.

**Table 1:** **Bacterial strains and plasmids used in this study.**

Strain or plasmid	Relevant characteristics	Sources
Strains		
*G. parasuis* SC1401	Serotype 11 clinical isolate (Sichuan, China), CDT-positive	Laboratory collection
*G. parasuis* SC1401 Δcdt	A CDT-deficient mutant strain, derived from SC1401, with both copies of the *CDT* gene completely deleted	Laboratory collection
Plasmids		
pET32a-cdtA	A 624 bp cdtA CDS in pET-32a ( +)	Laboratory collection
pET32a-cdtB	A 768 bp cdtB CDS in pET-32a ( +)	Laboratory collection
pET32a-cdtC	A 471 bp cdtC CDS in pET-32a ( +)	Laboratory collection

### Expression of *Gp*CDT

CDT is usually encoded by 3 genes present in operon 1, each encoding the CdtA, CdtB, and CdtC subunits, which bind together in a 1:1:1 ratio to form an active heterotrimeric holotoxin complex [27]. The pET32a-cdtA, pET32a-cdtB, and pET32a-cdtC plasmids were constructed and preserved in our laboratory. CdtA, CdtB, and CdtC were subsequently expressed according to the established induction conditions. His-tagged proteins were induced with 1.2 mM IPTG (MCE, China) for 16 h at 18 °C. CdtA, CdtB, and CdtC were mixed at a ratio of 1:1:1 and allowed to stand at 4 °C overnight [26].

### Cell treatment and infection

Coincubation of the *Gp*CDT protein with cells: 3D4/21 cells were inoculated into 6-well plates at a density of 1 × 10^6^ cells/well. *Gp*CDT (1 or 2 µg/mL) was diluted with serum-free RPMI 1640 (Gibco, USA) and coincubated with the cells for 48 h.

Bacterial infection of cells: Wild-type SC1401 and SC1401Δcdt cells were grown to logarithmic phase (OD = 0.6–0.8) and centrifuged at 5000 rpm for 2 min. After centrifugation, the supernatant was filtered through a 0.22 μm filter to remove live bacteria, and the supernatants were collected. Aliquots of 1 × 10^6^ 3D4/21 cells were added to 6-well plates with bacteria at multiplicities of infection (MOI) of 10 or 100 and incubated for 48 h.

After incubation, the cells were scraped into 5 mL centrifuge tubes and centrifuged at 3000 rpm for 5 min; the precipitates were collected for subsequent experiments.

Treatment of cells with ferroptosis inhibitors: 3D4/21 cells were inoculated in 6-well plates at a density of 1 × 10^6^ cells per well. DFO (MCE, China) and Fer-1 (MCE, China) were diluted to 50 μM and 5 μM, respectively, with serum-free RPMI 1640 medium, and 1 mL of inhibitor was added to each well and coincubated with the cells for 2 h. Finally, 2 μg/mL *Gp*CDT was added to the wells, and the mixture was incubated for 48 h.

### Cell viability assay

The cytotoxicity induced by *Gp*CDT was assessed with a CCK-8 kit (Beyotime Biotechnology, China). Briefly, 3D4/21 cells in 96-well plates were incubated with 10 μL of CCK-8 solution for 1 h. Absorbance was read at 450 nm with an enzyme labeller (Bio-Rad, USA). The cells were also observed by light microscopy, and images were captured (Olympus America, USA).

### Experimental animals

We used specific pathogen-free male C57BL/6 mice weighing 21 ± 1 g in this study. All of the animal experiments complied with the requirements of international experimental animal ethics (GB/T 35823–2018) and were approved by the Animal Ethics Committee of Sichuan Agricultural University (SYXK2019-187). After 1 week of adaptive feeding, the mice were randomly divided into three groups of three mice each: the control, wild-type SC1401, and SC1401Δcdt groups. At 6 weeks of age, the mice were dosed with bacteria by intraperitoneal injection (1 × 10^9^ CFU), and the control mice were injected with an equal volume of sterile 0.9% saline. The mice were sacrificed at 24 h post-infection, and the lungs were removed for subsequent experiments.

### Histopathology

The fixed mouse lung tissues were removed from 10% neutral formalin buffer (Bossci, China), trimmed flat, and then placed into numbered embedding frames. Dehydration was then performed by placing the embedding frames in a dehydrator, dehydrating them sequentially with different concentrations of alcohol, and then dipping them in wax. After that, the wax-impregnated tissues were embedded in the embedding machine, and the wax blocks were removed and trimmed after the wax solidified. Finally, the trimmed wax blocks were sliced on a paraffin slicer (Thermo Fisher Scientific, USA) with a thickness of 4 μm, and after slicing, the slices were baked on a tissue slicer (Changzhou Zonway Electronic Instrument Co., Ltd., China) at 60 °C for 30 min to 1 h.

For haematoxylin and eosin (H&E) staining, the fixed, dehydrated and embedded tissues were first cut into thin slices and pasted on slides, deparaffinized with xylene, and then hydrated with different concentrations of ethanol (100–70%). Then, the nuclei of the cells were stained with haematoxylin staining solution for 3‒8 min and washed to return to the blue colour after differentiation in ethanol hydrochloride, and the cytoplasm of the cells was subsequently stained with eosin staining solution for 1‒3 min. After that, the cells were dehydrated with different concentrations of ethanol (80‒100%), cleared with xylene, and finally, the slices were sealed with neutral gum for observation.

### Determination of oxidative stress-related indicators

In this study, a variety of kits (Solarbio, China) were used to determine the contents of ferrous ions (catalogue number: BC5415), GSH (catalogue number: BC1175), and MDA (catalogue number: BC0025). The specific procedures are as follows:

For tissue samples, approximately 0.1 g of mouse lung tissue was taken and homogenized with 1 mL of extraction solution in an ice bath. The homogenate was centrifuged at 10 000–12 000 × *g* for 4 min, and the supernatant was collected for subsequent analysis. For the cell samples, approximately 5 × 10^6^ cells were suspended in 1 mL of extraction solution, and then ultrasonic disruption was carried out. The disrupted cell suspension was centrifuged at 8000 × *g* for 10 min at 4 °C, and the supernatant was collected for subsequent analysis. The contents of ferrous ions, GSH, and MDA in the samples were subsequently determined in strict accordance with the instructions of the kits provided by the manufacturers.

### ROS measurement

The level of ROS was determined using a 2′,7′-dichlorofluorescin diacetate probe (DCFH-DA). (Beyotime, China). The culture medium was removed from the cells in a six-well plate, replaced with 1 mL of 10 μM DCFH-DA, and then incubated for 20 min at 37 °C. The cells were then washed three times with PBS, the fluorescence intensity was measured, and the level of ROS was determined as previously described [28].

### Quantitative real-time PCR

Cells from six-well plates were collected, and RNA was extracted using a UNIQ-10 Column Total RNA Purification Kit (Sangon, China), followed by two-step reverse transcription using a gDNA Eraser’s PrimeScript™ RT Reagent Kit (Takara, Japan). SYBR Premix EX Taq™ II (Tli RNaseH Plus; Takara, Japan) was used for real-time quantitative PCR (qPCR) of the transcripts. The primers used for qPCR (P3–P10) are listed in Table 2. Gene expression was quantified using the 2^−ΔΔCt^ method; the results are presented relative to the expression of β-actin. A Lightcycler96 (Roche, Switzerland) system was used for qPCR. For each sample, there were three biological replicates, and the qPCR assay was repeated three times.

**Table 2:** **Primers used in this study.**

Primers	Sequence (5′–3′)	Product (bp)
PCR primers		
P1(cdt-F)	AATTTGGAAAACTATACGG	2063
P2(cdt-R)	ACGTTTTTTTACAAAGCTG	
qPCR primers		
P3(GPX4-F)	CCATGCACGAATTCTCAGCC	97
P4(GPX4-R)	TGAGAGGCCACATTGGTGAC	
P5(ACSL4-F)	GTTCACGACAAGCCAAACCC	359
P6(ACSL4-R)	TGGGCCAAAAGTGTCAACCT	
P7(FTH-F)	AGCTCTATGCCTCCTACGTCT	342
P8(FTH-R)	GCTCATCCAGGTAATGCGTCT	
P9(β-actin-F)	CTTCCTGGGCATGGAGTCC	201
P10(β-actin-R)	GGCGCGATGATCTTGATCTTC	

### Western blotting

All the samples were lysed using RIPA lysis buffer (Beyotime, China). For tissue samples, 20 mg of tissue was added to 200 μL of lysis buffer and 2 μL of PMSF. For the cell samples, 150 µL of lysis buffer and 1.5 µL of PMSF were added to the cells in a 6-well plate. After sufficient lysis, the samples were centrifuged at 10 000–14 000 × *g* for 3–5 min, and the supernatants were collected. The samples were resolved by SDS‒PAGE and then transferred to PVDF membranes. The membranes were blocked with 5% skim milk and then incubated overnight at 4 ℃ with the following primary antibodies: rabbit anti-β-actin mAb (1:10 000), rabbit anti-GPX4 mAb (1:5000), rabbit anti-FTH mAb (1:1000) (ABclonal, China), or rabbit anti-ACSL4 mAb (1:2500, Proteintech, China). Following five washes with TBST, the membranes were incubated with HRP-conjugated goat anti-rabbit IgG (1:5000, BOSTER, China) for one hour at room temperature and then washed five more times with TBST. Proteins were visualized by using an enhanced chemiluminescence (ECL) reagent (Bio-Rad, USA).

### Statistical analysis

Statistical analysis was performed with GraphPad Prism version 6.0. Student’s *t* test, one-way analysis of variance (ANOVA), or two-way ANOVA was used to assess statistical significance. Significant differences between groups are denoted by *p < 0.05, **p < 0.01, ***p < 0.001, and ****p < 0.0001. The error bars in all the figures represent the standard deviation of three independent experiments.

## Results

### *Gp*CDT induces ferroptosis in 3D4/21 cells

**Figure 1:**
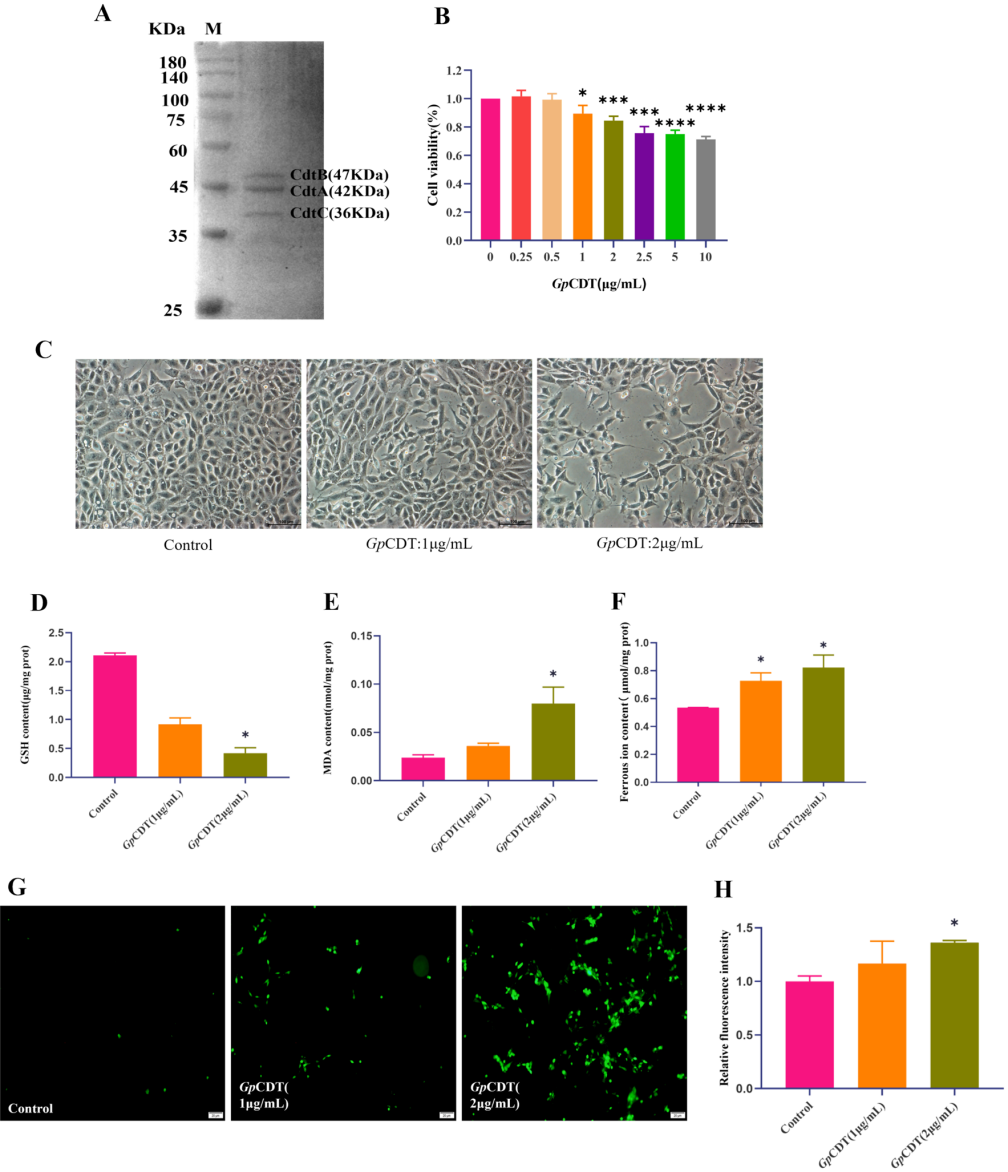
**Ferroptosis biomarkers in *Gp*CDT-treated cells.** **A** SDS‒PAGE of purified *Gp*CDT. **B** Cell viability, as measured by CCK-8, of 3D4/21 cells treated with *Gp*CDT for 48 h. Significant differences between groups are indicated by *p < 0.05, ***p < 0.001, ****p < 0.0001. **C** Typical morphology of 3D4/21 cells treated with *Gp*CDT for 48 h (magnification: 200 × ; scale bar: 100 µm). **D** Effect of *Gp*CDT on the intracellular GSH level (*p < 0.05). (E) Effect of *Gp*CDT on intracellular MDA levels (*p < 0.05). **F** Effect of *Gp*CDT on the intracellular ferrous ion concentration (*p < 0.05). **G**, **H** Effect of *Gp*CDT on intracellular ROS levels (*p < 0.05).

Figure 1A shows the purification of *Gp*CDT, which was resolved on a 12.5% SDS‒PAGE gel, indicating that the recombinant holotoxin was successfully prepared and could be used for the following experiments. As shown in Figure 1B, the viability of 3D4/21 cells treated with purified *Gp*CDT at a concentration of 1 µg/mL decreased significantly. When the toxin concentration exceeded 1 µg/mL, the cytotoxicity of *Gp*CDT occurred in a dose-dependent manner, indicating that the cell viability decreased significantly as the toxin concentration increased. Representative samples of control and *Gp*CDT-treated 3D4/21 cells are displayed in Figure 1C. The treated cells exhibited increased volume, uneven size distribution, and a notable reduction in cell number, with these effects becoming more pronounced at higher *Gp*CDT concentrations. To detect ferroptosis in *Gp*CDT-treated cells, key biomarkers of ferroptosis, including GSH depletion, lipid peroxidation, and iron accumulation, were further investigated. Figure 1D shows the decrease in GSH levels in treated cells, whereas Figure 1E shows the increase in the levels of MDA, a marker of lipid peroxidation. Figure 1F highlights the accumulation of unstable ferrous ions. To assess the degree of ferroptosis, intracellular ROS levels were measured. Figure 1G, H presents representative samples showing increased green fluorescence, indicative of elevated intracellular ROS levels, which correlate with increased *Gp*CDT concentrations.

**Figure 2:**
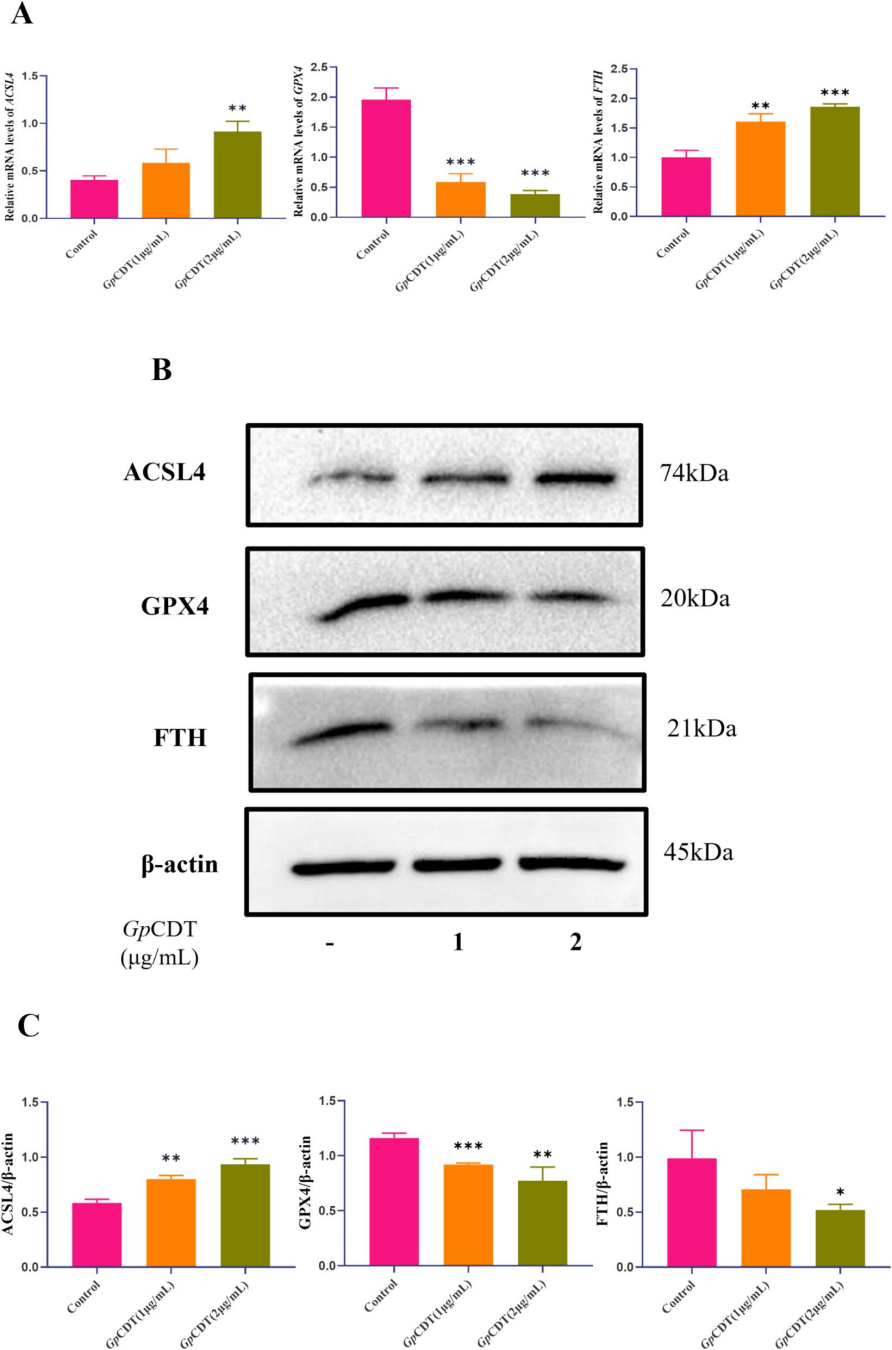
**Expression of ferroptosis-related genes and proteins in response to** ***Gp*CDT.** **A** Levels of *ACSL4*, *GPX4*, and *FTH* in *Gp*CDT-treated 3D4/21 cells as determined by RT‒qPCR. Significant differences between groups are indicated by **p < 0.01 and ***p < 0.001. **B**, **C** Representative western blot of ACSL4, GPX4, and FTH in *Gp*CDT-treated 3D4/21 cells. Significant differences between groups are indicated by *p < 0.05, **p < 0.01, and ***p < 0.001.

We simultaneously measured the transcript levels of the ferroptosis-related genes *ACSL4*, *GPX4*, and *FTH* and their expression levels (Figures 2A‒C). These assays demonstrated that treatment with *Gp*CDT resulted in a concentration-dependent decrease in the transcript levels of *GPX4* and an increase in the transcript levels of *ACSL4* and *FTH*. The changes in the protein expression of ACSL4 and GPX4 were consistent with the changes in gene transcription levels, whereas FTH expression decreased. We hypothesized from these results that *Gp*CDT leads to cellular ferritinophagy.

### Ferroptosis phenotypes are more pronounced in cells infected with SC1401 than in those infected with SC1401Δcdt

**Figure 3:**
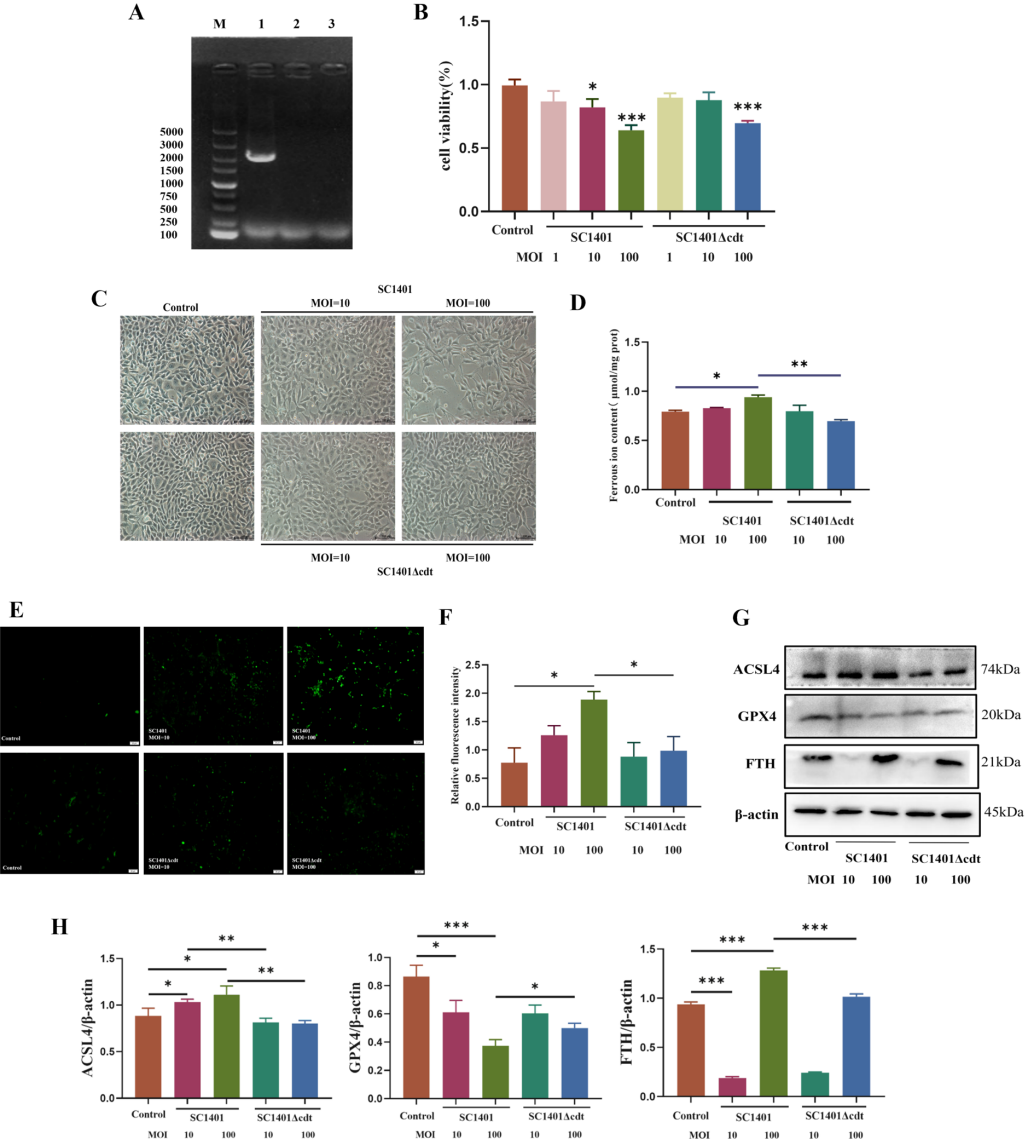
**Effects of infection with wild-type SC1401 or SC1401Δcdt *G. parasuis* on ferroptosis in 3D4/21 cells.** **A** PCR confirmation of the Δcdt mutant strain: (lane 1) wild-type *G. parasuis*, (lane 2) Δcdt *G. parasuis* and (lane 3) negative control. The primers used for PCR (P1–P2) are detailed in Table 2. **B** CCK-8 analysis of SC1401- and SC1401Δcdt-infected cells. Significant differences between groups are indicated by *p < 0.05 and ***p < 0.001. **C** Typical morphology of 3D4/21 cells infected with SC1401 or SC1401Δcdt *G. parasuis* for 48 h (magnification: 200 × ; scale bar: 100 µm). **D** Effects of SC1401 and SC1401Δcdt infection on ferrous ion concentrations (*p < 0.05). **E**, **F** Effects of SC1401 and SC1401Δcdt infection on the accumulation of ROS (magnification: 10×; scale bar: 20 μm; *p < 0.05). **G**, **H** Representative western blot of ACSL4, GPX4, and FTH in SC1401- and SC1401Δcdt-infected 3D4/21 cells (*p < 0.05, **p < 0.01, ***p < 0.001).

To further determine the role of *Gp*CDT-induced ferroptosis, we compared 3D4/21 cells infected with the SC1401 strain and SC1401Δcdt. Figure 3A shows the results of PCR amplification of the *CDT* region of the wild type and Δcdt *G. parasuis strains*, demonstrating the absence of the gene in the deletion strain. We evaluated the viability of 3D4/21 cells infected with SC1401 or SC1401Δcdt, and the results are shown in Figure 3B. Compared with the uninfected controls, both strains caused a significant decrease in viability at an MOI of 100. At an MOI of 10, only the wild-type strain exhibited a decrease in viability. To gain a deeper understanding of the microscopic changes in cells following infection by different strains, we observed the morphology of the infected cells, with representative images presented in Figure 3C. At an MOI of 10, compared with SC1401Δcdt cells, SC1401-infected 3D4/21 cells significantly expanded in size and became loosely arranged with a loss of their original morphology. Moreover, vacuoles of varying sizes formed, and cytopathic lesions were obvious. These effects were more pronounced at an MOI of 100. On the basis of these findings, we further investigated the levels of labile iron ions within the cells, which are closely associated with ferroptosis. The results are presented in Figure 3D and illustrate that the levels of unstable iron ions were greater at an MOI of 100 in SC1401-infected cells than in SC1401Δcdt-infected cells. Differences in bacteria did not affect subferric ions in cells at an MOI of 10. The ROS levels in SC1401-infected cells were significantly greater than those in SC1401Δcdt-infected cells (MOI of 100) (Figures 3E, F). To analyse the regulatory mechanisms of ferroptosis at the molecular level in detail, Figure 3G shows the levels of ACSL4, GPX4 and FTH determined by western blotting after infection with SC1401 and SC1401Δcdt. Infection with SC1401 significantly upregulated the protein expression level of ACSL4, whereas infection with SC1401Δcdt did not affect the protein level. Infection with SC1401 significantly decreased the expression level of GPX4, whereas infection with SC1401Δcdt at an MOI of 100 significantly increased the expression of GPX4 compared with that in SC1401-infected cells. FTH is a marker of disordered iron metabolism. The level of FTH decreased in SC1401-infected cells at an MOI of 10 but significantly increased at an MOI of 100. At an MOI of 100, the level of FTH was significantly lower in SC1401Δcdt-infected cells than in SC1401-infected cells (Figure 3H).

### SC1401 triggers ferroptosis in C57BL/6 mice

**Figure 4:**
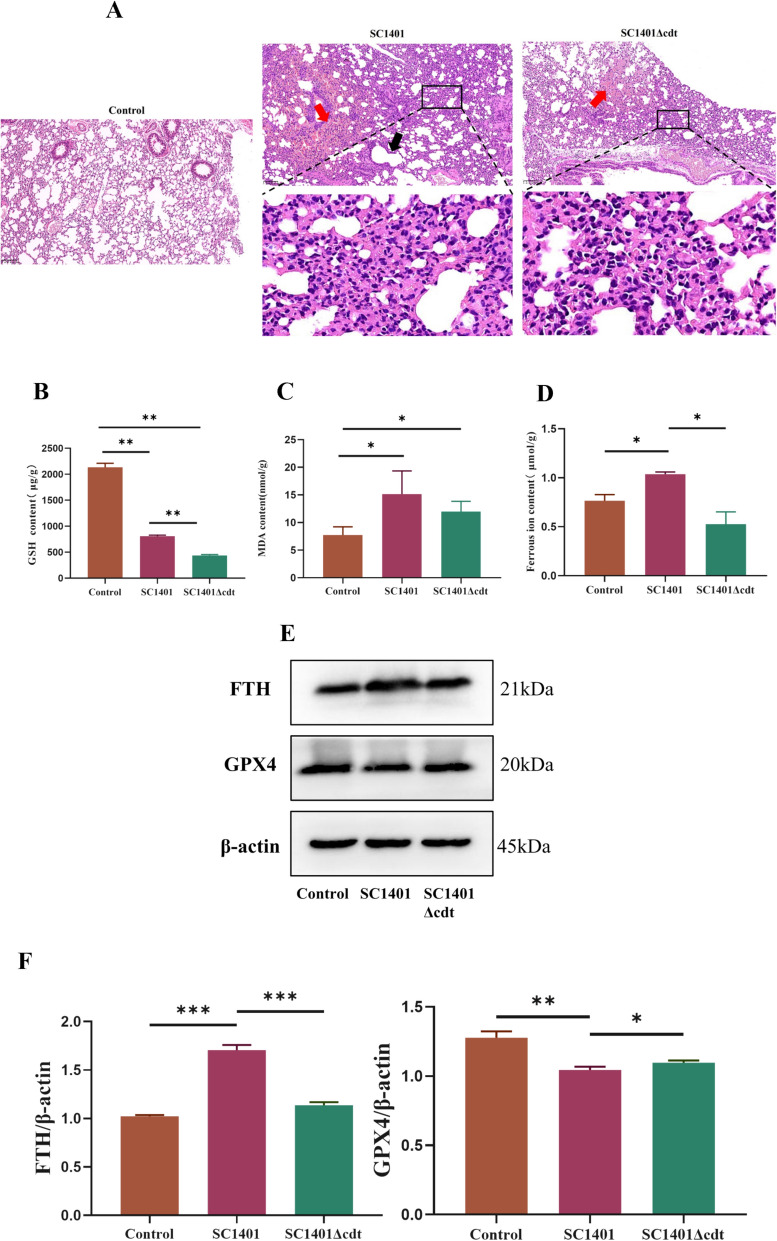
**Impact of *G. parasuis* infection on ferroptosis in mice.** **A** *G. parasuis* induced histopathological changes in mouse lungs. The black box indicates extensive solid lung lesions (magnification: 200 × , scale: 100 µm). **B** GSH levels in the indicated groups (**p < 0.01). **C** MDA levels in the indicated groups (*p < 0.05). **D** Ferrous iron content of the indicated groups (*p < 0.05). **E**, **F** Representative western blots of FTH and GPX4 in lung tissues (*p < 0.05, **p < 0.01, ***p < 0.001).

Having demonstrated, in vitro, the role of ferroptosis in cell death triggered by *Gp*CDT and *G. parasuis* SC1401, we next investigated whether the same mechanisms are involved in *G. parasuis*-induced ferroptosis in vivo. For this purpose, we infected mice with the SC1401 or SC1401Δcdt strains by intraperitoneal injection. Lung tissues were collected, and as shown in Figure 4A, histologic staining revealed normal morphology in the control group. Compared with those of the control group, the lung tissues of the mice infected with SC1401 had numerous pulmonary solid lesions (black boxes), capillary stasis in the alveolar wall with little neutrophil infiltration, focal haemorrhages (red arrows), and alveolar dilatation (black arrows). In the SC1401Δcdt-treated mice, the lung tissue displayed thickening of the alveolar wall with little neutrophil infiltration (black boxes) and focal haemorrhages (red arrows). The levels of GSH, MDA, and ferrous ions were also measured in these tissues (Figures 4B–D). Compared with control mice, SC1401- and SC1401Δcdt-infected mice presented significantly decreased levels of GSH and significantly increased levels of MDA. Only in the lungs of the SC1401-infected mice did the levels of ferrous ions significantly increase compared with those in the controls. These results are consistent with the characteristics of ferroptosis. Western blotting for FTH and GPX4 in lung tissues revealed that FTH was significantly increased and that GPX4 was significantly decreased by SC1401 infection. Compared with SC1401-infected mice, SC1401Δcdt-infected mice presented significantly lower levels of FTH and elevated levels of GPX4, which tended to be restored to control levels (Figures 4E and F).

### Inhibition of ferroptosis prevents *Gp*CDT-induced cell death

**Figure 5:**
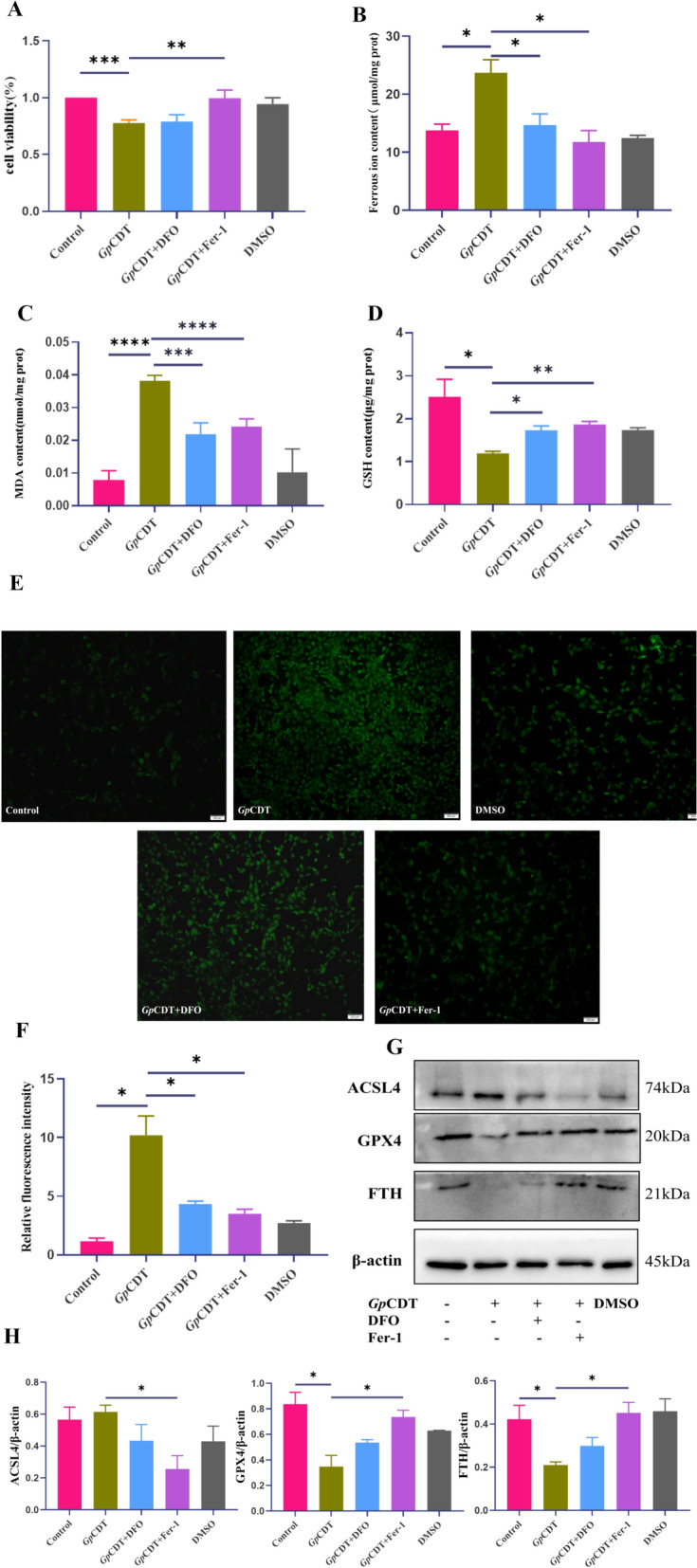
**Effects of ferroptosis inhibitors on multiple ferroptosis-related indicators in 3D4/21 cells.** **A** Cell viability assays. Significant differences between groups are indicated by **p < 0.01 and ***p < 0.001. **B** Ferrous ion content (*p < 0.05). **C** Determination of the MDA content (***p < 0.001, ****p < 0.0001). (D) Determination of the GSH content (*p < 0.05, **p < 0.01). **E**, **F** Intracellular ROS levels (*p < 0.05). **G**, **H** Western blot of ACSL4, GPX4, and FTH in 3D4/21 cells after treatment with ferroptosis inhibitors and *Gp*CDT (*p < 0.05).

To further investigate the effects of *Gp*CDT on lipid peroxidation and ferroptosis in vitro, we pretreated 3D4/21 cells with ferrostatin-1 (Fer-1) or deferoxamine (DFO). Figure 5A shows the results of the CCK-8 viability assays; Fer-1 pretreatment significantly attenuated *Gp*CDT-induced cell death, whereas DFO pretreatment had little effect, and because of the properties of DFO, we hypothesized that other modes of cell death were present in the cells at this time. The *Gp*CDT-induced increase in ferrous ion content was suppressed by Fer-1 and DFO pretreatment (Figure 5B). Pretreatment with Fer-1 and DFO also reduced intracellular MDA levels (Figure 5C) and increased the cellular antioxidant capacity, as characterized by elevated GSH levels (Figure 5D). The levels of intracellular ROS were lower in Fer-1- and DFO-pretreated cells than in untreated control cells (Figures 5E and F). In addition, Fer-1 inhibited the *Gp*CDT-induced upregulation of ACSL4, which was accompanied by increased expression of GPX4 and FTH. In DFO-pretreated cells, there were no significant differences in the levels of GPX4 or FTH compared with those in non-pretreated controls. We hypothesize that this finding is correlated with the cell viability status (Figures 5G and H). Although DFO does not significantly affect the expression of ferroptosis-associated proteins, it plays a role in antagonizing the *Gp*CDT-induced changes in the levels of GSH, MDA, and ferrous ions. Taken together, these results indicate that Fer-1 and DFO pretreatment attenuates *Gp*CDT-induced 3D4/21 cell death by ameliorating lipid peroxidation and restoring the antioxidant capacity of cells.

## Discussion

*G. parasuis* is one of the main causes of death in piglets aged 2 weeks to 4 months and is one of the main bacterial diseases that burdens pig farming industries worldwide [29]. Cytolethal distending toxin is a significant virulence factor of *G. parasuis* that exerts its toxic effects by inducing host DNA damage that often leads to cell cycle arrest and apoptosis [30]. A number of studies have explored the mechanism of CDT-induced cells in a variety of cell types. CDT can induce p53-dependent apoptosis in porcine respiratory epithelial cells [31]. CDT enters the cell by binding to cholesterol, engages in endocytosis pathways, and triggers apoptosis by causing DNA double-strand breaks through the DNase activity of its CdtB subunit [32]. Additionally, CDT can cause cellular pyroptosis, and some studies have shown that CDT induces pyroptosis through the ROS/caspase-9/caspase-3/GSDME signalling pathway [6]. To date, there are no reports of CDT causing other modes of cell death.

Here, we report that *Gp*CDT causes ferroptosis both in vivo and in vitro. In 3D4/21 cells treated with *Gp*CDT (2 µg/mL) or infected with SC1401 (MOI of 100), redox homeostasis in the host is disrupted, GSH is depleted, the production of ROS and the lipid peroxidation product MDA is increased, and large amounts of ferrous ions are produced. Changes in the expression of FTH and GPX4 further indicated the occurrence of ferroptosis. Inhibitors of ferroptosis, DFO and Fer-1, inhibit the production of ROS by regulating the protein levels of FTH, ACSL4, and GPX4, thereby alleviating the cellular damage caused by *Gp*CDT. Our results suggest that ferroptosis may be another mechanism by which *Gp*CDT exerts cytotoxicity.

Iron homeostasis in organisms is regulated at different levels; at the systemic level, iron homeostasis is regulated mainly by heparin, whereas at the cellular level, it is regulated mainly by iron metabolism-related proteins [33]. Iron exists in two forms in cell metabolism: ferrous cations (Fe^2+^) and ferric cations (Fe^3+^). Fe^3+^ binds to plasma transferrin in the blood and enters cells via transferrin receptor 1 (TfR1) on the cell membrane. Fe^3+^ is reduced to Fe^2+^ by six transmembrane epithelial antigen of prostate 3 (STEAP3) and then enters the cytoplasm through divalent metal transporter 1 (DMT1). Free Fe^2+^ in the cytoplasm is metabolically active and can participate in a series of intracellular enzymatic processes, including DNA synthesis and repair, the cell cycle, and the Fenton reaction. Fe^2+^ is transported out of the cell through ferroportin (FPN). Some of the intracellular Fe^2+^ is stored in the cytoplasm in the form of ferritin, including FTH and FTL. The other Fe^2+^ exists in the form of a pool of labile iron. An increase in the labile iron pool is prone to cause the Fenton reaction, leading to the accumulation of intracellular ROS and ultimately ferroptosis [34–36]. Ferritin is the storage form of free iron in cells; it is composed of FTH and FTL. FTH has ferroxidase activity and oxidizes ferrous iron to ferric iron, which can protect cells from oxidative stress damage [37]. Low levels of FTH in cells may lead to an increase in intracellular free iron, resulting in an increase in the labile iron pool and various modifications of DNA bases along with an increase in intracellular lipid peroxidation levels [38].

FTH acts as a negative regulator of ferroptosis; here, we found that FTH decreased with increasing *Gp*CDT concentration; however, its transcriptional level increased. We hypothesized that this is due to ferritin autophagic degradation mediated by *Gp*CDT activation of NCOA4 (nuclear receptor coactivator) in 3D4/21 cells. NCOA4 and FTH together maintain intracellular iron homeostasis [39]. NCOA4 is a selective receptor of ferritin; it binds FTH, and then the NCOA4-ferritin complex is translocated to the autophagic lysosome, which mediates the degradation of ferritin and the conversion of ferritin-bound iron to free iron, thereby inducing ferroptosis [40–42]. Mancias et al. reported that NCOA4 levels are tightly regulated by intracellular iron levels, and in iron-enriched cells, NCOA4 binding to HERC2, an E3 ubiquitin ligase, is increased, leading to proteasomal degradation of NCOA4. Decreased levels of NCOA4 inhibit ferritin autophagy and increase ferritin storage of iron [42]. As *Gp*CDT causes an increase in the number of intracellular iron ions, more FTH is needed to store the excess iron ions; as Fuhrmann et al. reported, FTH expression increases with increasing intracellular iron content [42]. This is likely the reason for the elevated level of FTH transcription we observed in the *Gp*CDT-treated cells. In response to ferritin autophagy, FTH is degraded, and its expression is reduced. Compared with Fer-1, DFO, an inhibitor of ferroptosis, did not significantly increase the expression of FTH. DFO mainly exerts its effects by chelating iron ions and reducing iron-dependent lipid peroxidation, although it has been reported to act as an inducer of apoptosis [43]. We hypothesized that the apoptotic pathway is also involved in *Gp*CDT-induced 3D4/21 cell death and that the process of *Gp*CDT-induced apoptosis was accelerated due to the actions of DFO. In the SC1401 vs SC1401Δcdt experiments, the expression levels of FTH appeared abnormal. When the number of bacteria was relatively small (MOI of 10), CDT secreted by SC1401 caused ferroptosis in 3D4/21 cells, which led to a decrease in the expression of FTH. The infecting bacteria need iron for their growth and reproduction and secrete iron carriers to meet these needs [44]. This leads to an imbalance in intracellular iron homeostasis, resulting in a massive release of iron ions originally stored in the FTH to meet bacterial growth requirements. This may explain why SC1401Δcdt (MOI of 10) infection of 3D4/21 cells also limits FTH expression. When the number of infecting bacteria is high (MOI of 100), a strong inflammatory response is triggered. The release of inflammatory cytokines such as interleukin-6 (IL-6) affects FTH proteins. IL-6 stimulates cells to increase the synthesis of FTH proteins to store more iron ions, which is a cellular defence strategy to prevent bacteria from acquiring too many iron ions [45]. Although SC1401Δcdt does not induce ferroptosis, the iron carriers secreted by bacteria bind to intracellular iron ions and “hijack” the iron in ferritin, altering the original iron storage state of ferritin and leading to the disruption of cellular ferritin expression [46]. When bacteria invade an organism, the host’s immune system initiates a series of responses to limit the pathogen’s iron acquisition [47]. We hypothesized that in SC1401-infected mice, even when ferroptosis occurred, the lack of decrease in FTH expression was due to a series of host immune responses. In contrast, SC1401Δcdt does not trigger such a strong immune response due to the absence of CDT, an important virulence factor, and significantly reduced FTH expression in infected mice. In summary, *Gp*CDT may accelerate the ferroptosis process by interfering with iron metabolism in the host and disrupting the storage and oxidative stress state of iron ions.

We investigated the effects of *Gp*CDT on ferroptosis-related pathways, including lipid, amino acid, and glutathione metabolism pathways. System XC-(xCT) is an amino acid reverse transporter protein composed of two subunits, SLC7A11 and SLC3A2. Located on the cell membrane, xCT exchanges cystine and glutamate inside and outside the cell at a 1:1 ratio; the cystine entering the cell is reduced to cysteine, which is catalysed by cystine reductase [48]. GSH is subsequently synthesized in the presence of γ-glutamylcysteine synthetase and glutathione synthetase [49]. Glutathione can cycle between the reduced (G-SH) and oxidized (G-S–S-G) states, allowing it to participate in intracellular redox reactions [50]. Glutathione peroxidase (GPX) uses GSH as a substrate to reduce toxic lipid peroxides to nontoxic lipids and alcohols, thereby protecting the cellular structure and function from peroxide damage. Several GPx family members, including GPX1-GPX8, have been identified in mammals. However, GPX4 is the only protein that converts GSH into GSSG and reduces toxic lipid peroxides to nontoxic lipid alcohols [51]. Therefore, GPX4 has important implications for the removal of lipid peroxides and the prevention of ferroptosis. Here, we found that after *Gp*CDT treatment, 3D4/21 cells presented a significant decrease in GSH content and GPX4 levels, indicating that intracellular oxidative homeostasis was disrupted; this phenomenon could be counteracted by pretreatment with the ferroptosis inhibitors DFO and Fer-1. However, the effect of DFO was not as significant as that of Fer-1; as a result, the DFO-treated cells were not as vigorous as the Fer-1-treated cells and were not able to maintain their redox balance sufficiently. GPX4 protein expression was significantly decreased in SC1401-infected cells, but we obtained similar results in SC1401Δcdt cells. After bacterial invasion of cells, a series of intracellular stress responses are triggered, which change the intracellular metabolic environment and lead to excessive production of reactive oxygen species in the cells. Cells regulate the expression of a series of antioxidant proteins to cope with the sharp increase in reactive oxygen species, and in this process, the expression and stability of the GPX4 protein are affected [52]. Although SC1401Δcdt lacks the *CDT* gene, bacterial virulence factors are rich in variety, diverse in function, and cause a stress response in the cell. In animal experiments, GPX4 expression levels were also downregulated by SC1401 infection, and these alterations were highly correlated with ferroptosis.

In addition to being essential components of cell and organelle membranes, lipids regulate various biological functions [53]. Different types of lipids regulate different types of biological processes. Free polyunsaturated fatty acids (PUFAs) react with phosphatidylethanolamine (PE) in the presence of ACSL4 and LPCAT 3 to synthesize phospholipids containing PUFAs (PUFAs-PEs) [54]. ACSL4 is a key enzyme in the regulation of lipid composition, especially through the regulation of PE. ACSL4 promotes lipid peroxidation, which in turn contributes to ferroptosis; it catalyzes the preferential oxidation of PEs containing arachidonic acyls and adrenaline acyls, which is the main cause of ferroptosis [55]. We found that the expression of ACSL4 in cells treated with *Gp*CDT or infected with SC1401 was significantly increased but could be antagonized by Fer-1, indicating that *Gp*CDT leads to ferroptosis in 3D4/21 cells. These results suggest that disruption of GSH metabolism (which is triggered by increased expression of ACSL4, suppressed expression of GPX4 and lipid peroxidation) is a crucial factor in *Gp*CDT-induced ferroptosis in the host.

In conclusion, our experiments revealed that, in addition to inducing apoptosis and pyroptosis, CDT causes cell death via ferroptosis. We investigated *Gp*CDT-induced ferroptosis in vitro via 3D4/21 cells treated with *Gp*CDT or infected with *G. parasuis* wild-type SC1401 or SC1401Δcdt. In vivo, we used C57BL/6 mice infected with *G. parasuis* wild-type SC1401 or SC1401Δcdt. In all cases, the levels of GSH, MDA, Fe^2+^, and ROS production, as well as the transcript levels and protein expression of GPX4, ACSL4, and FTH, were determined to establish that *Gp*CDT causes host lipid peroxidation, resulting in oxidative damage that leads to ferroptosis. Next, we will focus on ferritinophagy. However, whether ferritinophagy is involved in *Gp*CDT-induced ferroptosis and the role of ferritinophagy in this process have not been elucidated. Specifically, we investigated whether the induction of host ferroptosis by CDT necessitates the involvement of the entire toxin or is predominantly attributed to the core function of individual subunits. We intend to delve into this issue in the future. Investigating the mechanism of *Gp*CDT-induced ferroptosis in cells is conducive to understanding the mechanism by which *Gp*CDT exerts cytotoxicity and provides a foundation for a comprehensive analysis of the pathogenic mechanism of *G. parasuis*.

## Data Availability

All data supporting this study are included in this article.
